# A Liquid Metal‐Embedded Sheath‐Core Fiber with Internal Helical Structure for Strain‐Insensitive Electronics

**DOI:** 10.1002/advs.202509547

**Published:** 2025-07-21

**Authors:** Mengying Luo, Wanru Wei, Qiye Guo, Weibing Zhong, Kangyu Jia, Kangqi Chang, Ying Lu, Mufang Li, Dong Wang

**Affiliations:** ^1^ Key Laboratory of Textile Fiber and Products Ministry of Education Wuhan Textile University Wuhan 430200 China; ^2^ Institute of Technology for Future Industry Shenzhen Institute of Information Technology Shenzhen 518172 China

**Keywords:** elastic and conductive fiber, internal helical channel, liquid metal, strain‐insensitive conductor, wet‐spinning

## Abstract

Stable conductivity is crucial for flexible wearable devices, ensuring reliable signal transmission, sensing accuracy, optimal display performance, and overall device reliability. However, simultaneously achieving high elasticity, superior conductivity, and robust stability remains a formidable challenge. This study presents a novel core‐sheath fiber with an internal helical structure as the core layer and an intrinsic elastic material as the sheath layer, which combines the intrinsic elastic material and extensile spiral structure to realize high stretchability, ultra‐conductivity, and strain‐insensitivity. The hollow fiber with helical channel is fabricated via coaxial wet‐spinning technology by adjusting the flow velocity, with an elongation at break of ≈1440%. Subsequent infusion of liquid metal into the channel endows the fiber with outstanding conductivity, reaching 1.94 × 10^5^ S m^−1^. Benefiting from the helical structure, the obtained fibers exhibit outstanding strain‐insensitivity with a high Q value of 62.5 (resistance variation <1.6%) under 100% strain and show only 30% resistance change even at 600% elongation. The fibers exhibit superior stability against bending, twisting, and compressive deformations. The PU sheath provides excellent waterproof properties, enabling reliable operation in aqueous environments. Moreover, these fibers can be woven into fabrics, exhibiting outstanding performance in joule heaters, near‐field communication, and wireless charging applications.

## Graphical abstract

A stretchable fiber embedded with helical channels is fabricated via coaxial wet spinning. By filling liquid metal into the channels, a composite conductive fiber is constructed, featuring a helical conductive liquid metal core and an intrinsic elastic sheath. The integration of an intrinsic elastic matrix and an extensible helical conductive path enables the simultaneous realization of high stretchability, excellent conductivity, and strain insensitivity.

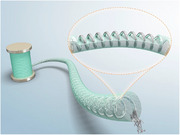

## 1 Introduction

Flexible conductive materials with ultra‐stretchability and robust conductivity have gathered extensive attention due to their advantages for deformable smart wearable electronics.^[^ ^1^, ^2^, ^3^, ^4^, ^5^ ^]^ Currently, stretchable and lightweight conductors are the development tendency of wearable electronics, among which fibers and textiles have attracted great research interest due to their advantages of softness, lightweight, ease of weaving, breathability, and strong wearability.^[^ ^6^, ^7^, ^8^, ^9^ ^]^ Conventional approaches to fabricating flexible conductors include blending, coating, filling, or in situ polymerization of conductive materials with elastic substrates.^[^ ^10^, ^11^ ^]^ However, they suffer from material delamination or conductivity degradation under strain. While resistive strain sensors effectively utilize this characteristic, they fail to meet the critical requirement for conductors to maintain nearly constant electrical resistance under deformation in applications such as power transmission wires, near‐field communication (NFC) antennas, and wireless charging systems. Thus, the design and fabrication of deformation‐insensitive and stretchable conductors are significant to developing flexible electronics.^[^ ^12^, ^13^, ^14^, ^15^ ^]^

Stretchable geometries such as kirigami (paper‐cutting art),^[^ ^16^ ^]^ folds,^[^ ^17^ ^]^ network structures,^[^ ^18^ ^]^ serpentine^[^ ^19^ ^]^ and helical structures^[^ ^20^, ^21^, ^22^ ^]^ have been utilized to fabricate stretchable conductive devices. The kirigami‐based materials are predominantly utilized without a substrate, which is not beneficial for recovery to the original shape after stretching.^[^ ^2^ ^]^ Whereas, the folding, network, and winding structures are generally used in combination with substrates. The interfacial adhesion and mechanical mismatch between the conductive materials and the substrates will lead to a decrease in stretchability and conductivity.^[^ ^4^ ^]^ Structural‐based stretchable conductors typically rely on out‐of‐plane deformation. However, subsequent processing steps such as encapsulation and integration can undermine their performance.^[^ ^2^ ^]^ Among these, the helical structure stands out as one of the most promising extended structures for stretchable electrical devices. Common approaches for fabricating helical fibers include wet‐spinning, buckling, twisting, and 3D printing. However, buckling and twisting often lack precise control over microscopic helical morphology and face challenges in achieving large‐scale production. The 3D printing suffers from slow processing speeds and high cost.^[^ ^23^ ^]^ In comparison, wet spinning provides exceptional structural homogeneity, precise controllability, and continuous processing capabilities, making it a more practical choice for scalable manufacturing. Furthermore, the wet spinning end products are fibrous materials with wearability, enabling direct integration into wearable applications. The present approaches utilize the helical structure to achieve flexibility and insensitivity of conductive materials, which have been widely reported and applied in wearable devices.^[^ ^24^, ^25^ ^]^ But usually, they have a direct helical structure and their conductive layers are exposed outside, which makes them susceptible to ambient temperature and humidity, and not beneficial for weaving. In addition, the conductive materials easily fall off the substrate, resulting in decreased conductivity during the straining process. Although these problems can be partially solved by encapsulating the conductive materials, Lee et al.^[^ ^26^ ^]^ wound PU fibers into a helical structure, loaded AgNPs on their surfaces, and then encapsulated them with PDMS to obtain composite spiral fibers. Tee et al.^[^ ^27^ ^]^ utilized a soft elastomer to encapsulate the spiral copper wire, improved the interface bonding issue with the adhesive, and realized integration with the sensing device. However, they still have shortcomings such as large device size, insufficient elasticity, and unstable helical structure during integration.

Recently, liquid metal has attracted great attention and has been gradually applied in the field of stretchable flexible electronics, due to its high conductivity, good fluidity, and deformation ability.^[^ ^28^, ^29^, ^30^, ^31^, ^32^, ^33^, ^34^, ^35^ ^]^ However, liquid metal‐based flexible devices fabricated through coating, blending, or printing often suffer from leakage issues and unstable performance. While directly infusing liquid metal into hollow fibers can address critical issues such as leakage and oxidation, this approach inevitably introduces conductivity instability due to Pouillet's law, where resistance varies with dimensional changes during mechanical deformation. Therefore, the design and fabrication of stretchable conductive materials with super stretchability, high conductivity, and ultra‐high stable conductivity still face significant challenges.

In this work, we developed a continuous ultra‐stretchable polyurethane‐based hollow fiber with an internal helical channel (HCF) via coaxial wet‐spinning. The helical structure could be controlled by spinning parameters such as needle size, spinning speed, and solution concentration. The fiber exhibits an extraordinary elongation at break of ≈1440%. The helical microchannel was filled with eutectic gallium‐indium (EGaIn) liquid metal (LM), forming a continuous conductive core within the sheath fiber that achieved a high conductivity of 1.94 × 10⁵ S m^−1^. Benefiting from the extensibility of the helical structure, the helical conductive path of the liquid metal mainly involves the unfolding of the helix. It remains essentially unchanged within a certain stretching range. The fiber exhibits a slight 1.6% change in electrical resistance at 100% strain. Moreover, this fiber can be widely applied in flexible electric devices and exhibits excellent performance in joule heaters, NFC, and wireless charging.

## 2 Results and Discussion

**Figure 1:**
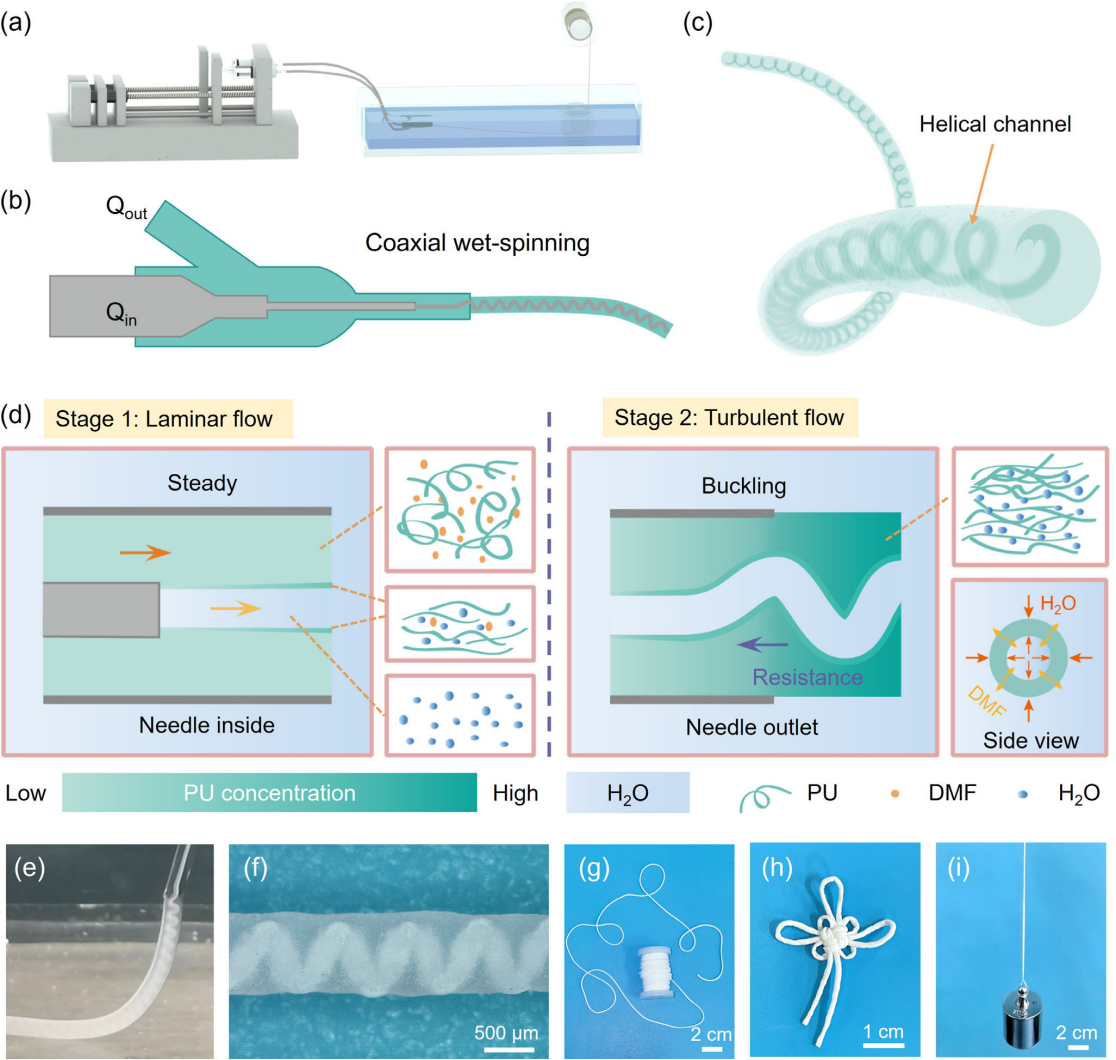
a) Schematic illustration of the coaxial wet‐spinning, b) Detail drawing of the coaxial needle, c) The prepared fiber with helical hollow structure, d) Scheme for the proposed mechanism of helical channel formation, e) Photograph images of the fiber generating process, f) Optical image of HCF, g) Photograph of a continuous HCF connected on a spindle, h) A Chinese knot made using HCF, i) HCF holding 200 g weight.

The hollow fiber with a helical channel structure was fabricated using coaxial wet‐spinning technology. As depicted in **Figure** **1**a, the assembled spinning apparatus primarily consists of three key components: a propeller, a coaxial needle, and a coagulation tank. The schematic diagram of the coaxial wet‐spinning is illustrated in Figure 1b and the photo image is in Figure S1a (Supporting information); the polymer spinning solution is in the outer channel, and the coagulating bath is extruded through the inner channel simultaneously, which differs from the preparation process of conventional helical fiber.^[^ ^36^, ^37^, ^38^ ^]^ A fiber with a helical channel could be achieved by improving the flow rate ratio (Q_out_/Q_in_) as depicted in Figure 1c. It is known that the fiber formation mechanism of wet spinning is based on double diffusion. When the coagulation bath in the needle tube comes into contact with the spinning solution, the molecules of the coagulation bath diffuse outward, and the solvent in the spinning solution diffuses inward. With continuous diffusion, the concentration of the spinning solution attains the critical concentration, thereby inducing in‐phase separation and leading to fiber solidification. To more effectively observe the formation process, a transparent coaxial needle was constructed using a capillary and a steel needle, as presented in Figure S1b (Supporting Information). Intriguingly, we noticed that the helical structure consistently emerged at the needle nozzle, as depicted in Figure 1e and Video S1 (Supporting Information). The fiber completes the preliminary forming within 5 s and continues to soak in the coagulation bath for 2 h to achieve complete solidification. It is inferred that in this spinning system, there is a double diffusion in the outer tube once the internal coagulator meets with the outer spinning solution. Nonetheless, it is insufficient to reach a critical concentration, which corresponds to stage 1 in Figure 1d. Until they enter a massive and static coagulating bath, two main shear stresses opposite the flow direction are generated. One is the internal frictional force (viscous drag) generated between the flowing fluid (spinning solution) and the stationary fluid (coagulating bath), which impedes the relative motion of the flowing fluids. The other is the structural resistance resulting from the solidification‐phase transformation when a large amount of coagulant diffuses into the spinning solution, causing the spinning solution to reach the critical concentration and solidify rapidly. These two forces lead to interfacial instability, thereby forming a helical structure, which corresponds to stage 2. We have also observed that external forces could generate the helical channel. In Video S2 (Supporting Information), the HCF was fabricated in a long container. As the fiber continuously grows in length, the polymer at the outlet is subjected to greater gravity, and thus, it cannot provide sufficient reverse force to make it fold; consequently, the channel becomes straight. When the fiber touches the bottom or the wall of the container, there is an upward supporting force, which can result in the fiber channel folding again. The appearance of the helical structure in the fiber can also be explained by Kelvin–Helmholtz instability, which refers to the instability occurring at the interface between two horizontally parallel streams with different velocities and densities.^[^ ^39^ ^]^ In the optical image presented in Figure 1f, it is evident that the fiber has an obvious helical channel structure. A continuous HCF was easily prepared as shown in Figure 1g, indicating its potential for large‐scale production. Figure 1h–i displays a knotted fiber and a fiber with a hang weight of 200 g, exhibiting its excellent flexibility and strength. Using a 30/21 G coaxial needle gauge enables the fabrication of the HCF with a smaller diameter, ≈300 µm, which is comparable to the sewing thread, as shown in Figure S3 (Supporting Information).

**Figure 2:**
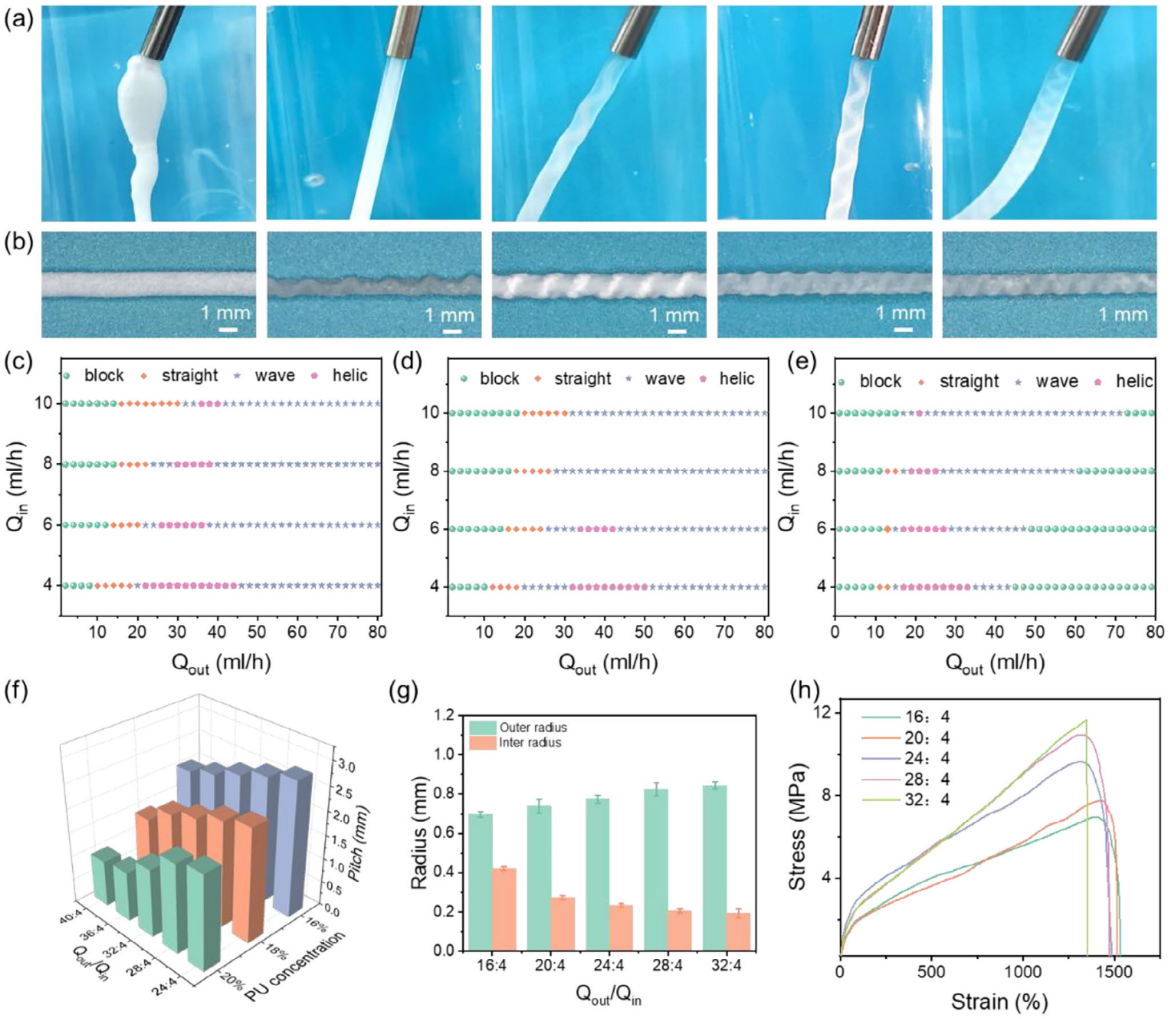
a) Photo images and b) Microscopy images of the fiber typical morphology at different Q_out_/Q_in_ with needle size 30/14, c) C_PU_ = 20 wt.%, d) C_PU_ = 18 wt.%, e) C_PU_ = 16 wt.%, f) Effect of PU concentration and Qout/Qin on the pitch with Q_in_ = 4 mL h^−1^, needle size 30/14, g) Relationship between the radius of the fiber and Q_out_/Q_in_, needle size 30/14, C_PU_ = 20 wt.% h) Tensile strain–stress curves of the fibers at different flow rates, needle size 30/14, C_PU_ = 20 wt.%.

The flow velocity plays a critical role in the formation of the helical channel fiber; the outer and inner flow speeds can regulate the internal structure of the fiber. As demonstrated in **Figure** **2**a and S2 (Supporting Information), systematic experimental investigations reveal a distinct progression of structural transformations under controlled flow conditions. When maintaining a constant inner flow rate, incremental increases in the external flow rate induce sequential morphological transitions within the internal structure of the fiber. This flow‐dependent evolution manifests as five distinct structural phases: initial flow blockage, followed by the development of a straight channel, which subsequently transforms into a large‐wave channel configuration. Further increases in external flow rate lead to the emergence of a helical channel structure, ultimately progressing to a dense wave channel pattern at higher flow differentials. Figure 2b shows the morphology images of dried HCF fabricated by the different flow rate ratios (Q_out_/Q_in_ = 16:4, 20:4, 24:4, 28:4, and 32:4). It can be found that the diameter of the fiber decreases after drying, and the pitch decreases gradually as the flow rate increases. The morphologies of the fiber structure are summarized in Figure 2c–e. It can be observed that the higher the flow rate ratio, the more readily a folded structure is formed. Additionally, compared to a lower PU concentration, a higher PU concentration facilitates the formation of a folded structure more easily. This phenomenon can be attributed to the enhanced solution viscosity at elevated PU concentrations (Figure S3, Supporting Information), which generates increased viscous resistance. The impacts of flow velocity and concentration on the helical pitch were investigated, as illustrated in Figure 2f. When the Q_out_/Q_in_ increases, or the PU concentration increases, the helical pitch of the fiber decreases. The higher Q_out_/Q_in_ and higher concentration generate a larger shear force, leading to a smaller pitch and amplitude. The influence of the Q_out_/Q_in_ on the sectional area of the whole fiber and its hollow part was analyzed, as depicted in Figure 2g. When the external phase flow rate gradually increases, the mass transfer flux increases, thus resulting in a larger fiber diameter. Nevertheless, the inner space will be crowded out, and then the hollow area and the helical amplitude will be reduced. This phenomenon is further corroborated by the images shown in Figure S4 and Video S3 (Supporting Information). Using 16 wt.% PU or 30/14G coaxial needles to prepare HCF under different Q_out_/Q_in_, the fiber structures also follow this principle as presented in Figures S5 and S 6 (Supporting Information). It can also be observed that as the PU concentration decreases, the fiber diameter decreases slightly. The helical channel fibers exhibit good mechanical properties, as shown in Figure 2h. The strain strength of the hollow fiber (from straight to helical channel) increases from 6.9 to 11.7 MPa owing to the larger amount of PU with the increase of Q_out_/Q_in_. The elongation at break is ≈1340%–1430%, which shows no obvious variation in regulation. Figure S7a,b (Supporting Information) exhibit a hysteresis loop in strain–stress curves of HCF under different strains. Cyclic stretching at 100% strain for 100 cycles, as presented in Figure S7c (Supporting Information), exhibits good anti‐fatigue properties.

**Figure 3:**
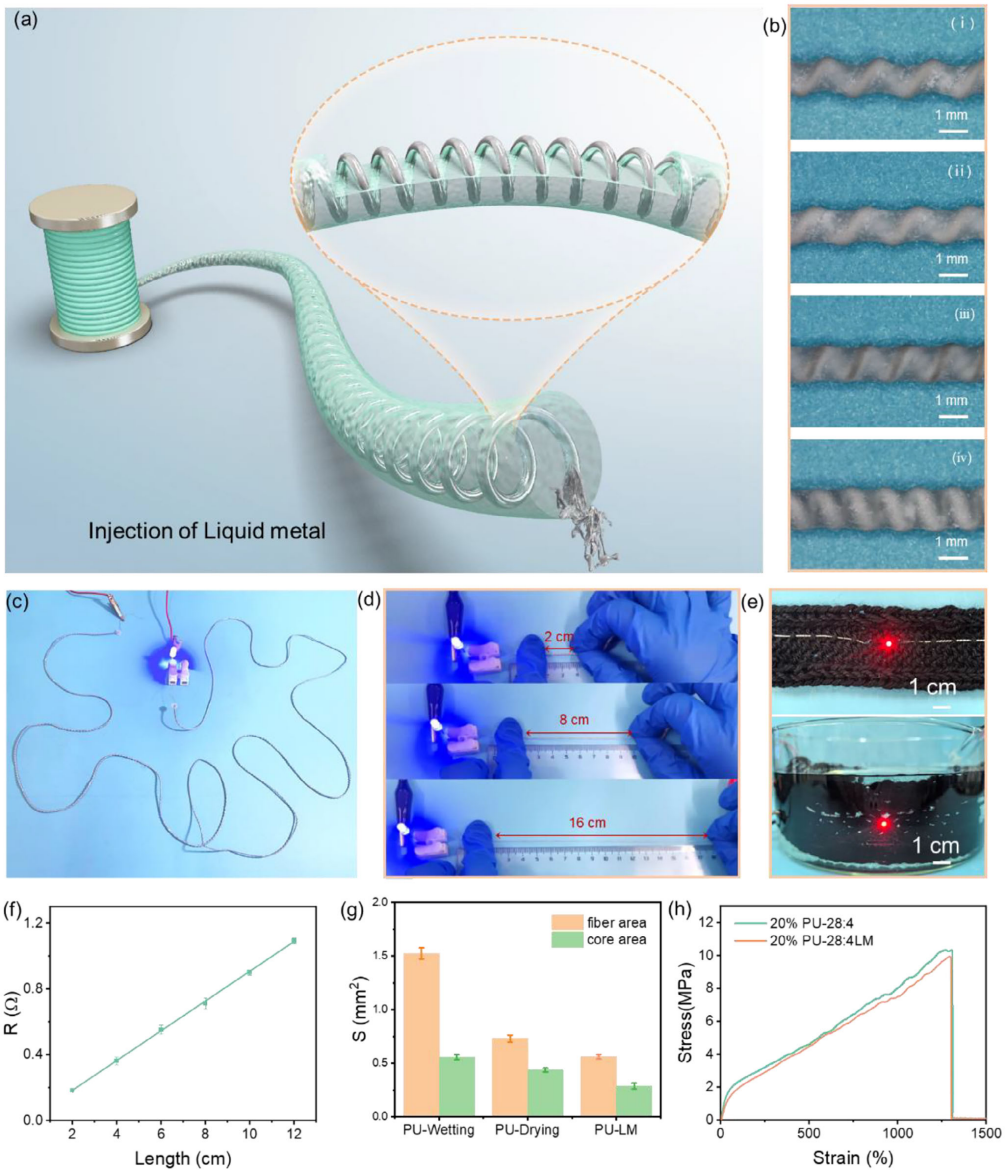
a) Schematic diagram of LM‐HCF, b) microscope images of LM‐HCF, Qout/Qin, i) 20:4, ii) 24:4, iii) = 28:4, iv) 32:4, c) A 50 cm LM‐fiber used as a connector for LED lighting, Qout/Qin = 28:4, needle size 30/14, d) Photograph images of LM‐HCF for LED lighting under stretching, f) Electrical resistance per length of LM‐HCF, g) Stress–strain curves of HCF and LM‐HCF. h) The sheath and core areas of HCF are in the wetting state, drying state, and LM‐filled state. Qout/Qin = 28:4, needle size 30/14.

The fiber was endowed with high conductivity by injecting LM, as exhibited in **Figure** **3**a. The microscopy images of the fibers are presented in Figure 3b, where the helical structure is visible. To better observe the internal helical structure, the fiber was immersed in dichloromethane to render it transparent. A more straightforward helical path of liquid metal is shown in Figure S8 (Supporting Information). To study the barrier property of polyurethane (PU) against liquid metals, a 25 cm LM‐HCF was subjected to pressing, twisting, and stretching tests, and its morphologies were continuously recorded for 10 days. As shown in Figure S9 (Supporting Information), the fiber surfaces remained clean without obvious liquid metal leakage. Video S4 (Supporting Information) presents the detailed process, demonstrating that wiping the fiber surface left the tissue completely clean, further confirming no liquid metal seepage. These results highlight the excellent barrier property of the PU layer. An LED could be lit using a 50 cm fiber as a connector with a small voltage of 2.3 V, as shown in Figure 3c. Figure 3d and Video S5 (Supporting Information) illustrate that the fiber can be stretched from 2 to 16 cm (700% strain) and still effectively light the LED, signifying its extremely stable electrical conductivity. Even when woven into a fabric and immersed in water, as shown in Figure 3e, the LED maintained a stable brightness, indicating that the PU layer provides good water resistance to the core layer. The fiber has a uniform and extremely low resistance of 0.092 Ω cm^−1^ (Figure 3f), equivalent to the electrical conductivity of 1.94 × 10^5^ S m^−1^. The cross‐sectional SEM image of HCF and LM‐HCF is shown in Figure S10 (Supporting Information), the LM‐HCF sheath and core areas of LM‐HCF are measured by Image J as shown in Figure 3g. Upon drying, the cross‐section of the fiber contracts. Moreover, the cross‐section of the fiber with liquid metal is smaller than that of the fiber without liquid metal. This can be attributed to the fact that during the hanging‐drying process, the fiber with liquid metal experiences a more substantial influence from gravity. Consequently, the fiber undergoes a certain extent of longitudinal stretching, leading to a decrease in its cross‐sectional area. The fiber elongation at break remains at ≈1300% after injecting LM, and the tensile strength decreases negligibly, as presented in Figure 3h, demonstrating its outstanding elasticity and strength.

**Figure 4:**
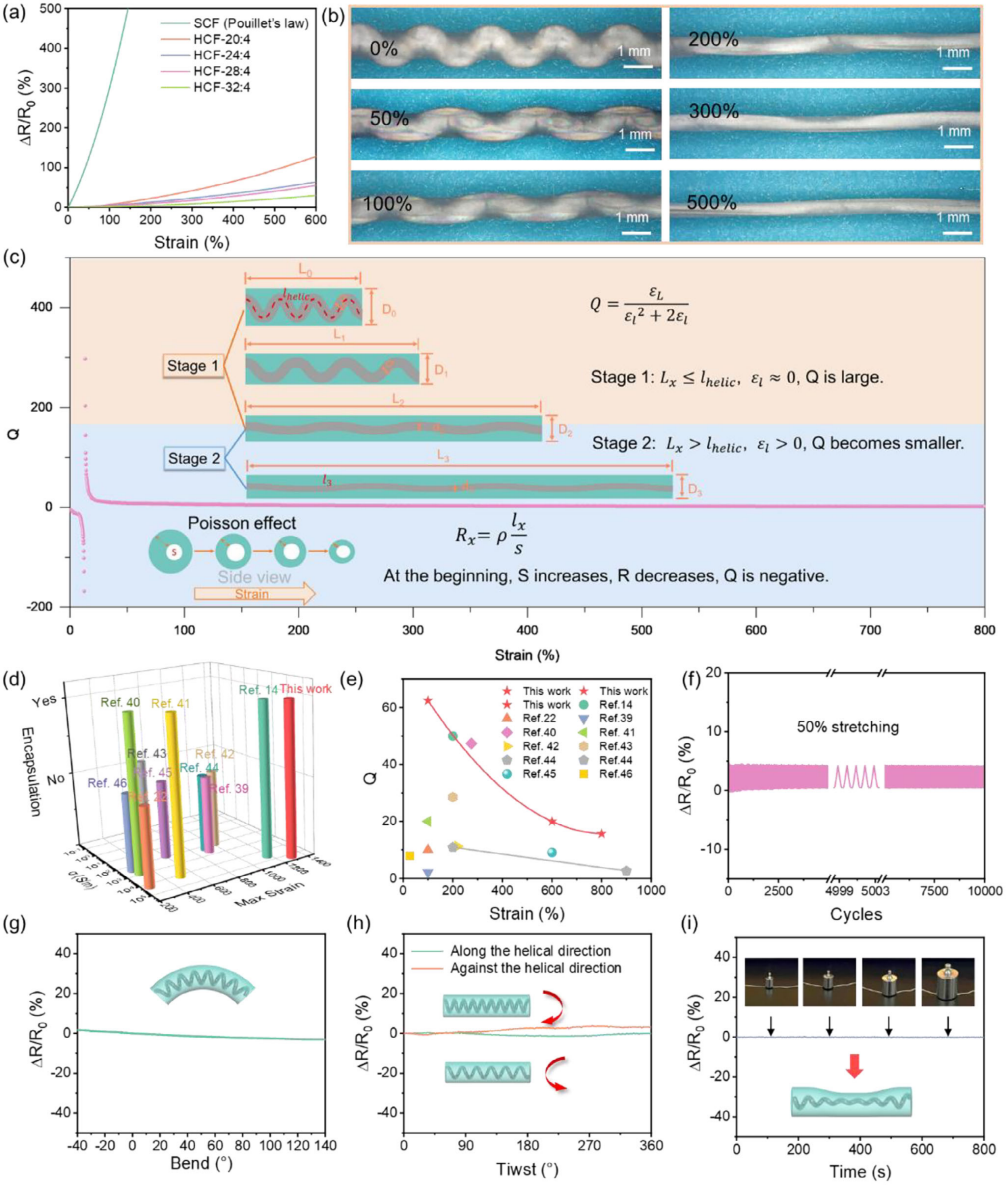
a) Stain‐dependent resistance changing rate of LM‐HCF, b) Microscope images of LM‐HCF helical change under different strains, c) Q values under different strains and the schematic diagram of the strain‐insensitivity mechanism, d) Comparison of the max strain and conductivity of the LM‐HCF with the data in reported literatures, e) Comparison of the Q value of the LM‐HCF with the data in reported literatures, f) Resistance change rates of the LM‐HCF under 700 cycles of 50% strain, g–i) Resistance change rates of LM‐HCF upon 5 times bending, 5 times twisting, and pressing.

To evaluate the conductive stability of LM‐HCF, the relative resistance change rate Δ*R*/*R*_0_ is used as shown in **Figure** **4**a. According to Pouillet's law, the LM‐filled fiber with a straight channel has a high strain resistance sensitivity. For LM‐HCFs, the stable electrical conductivity is attributed to the helical structure of the conductive path, which can be extended under continuous stretching. The conductive path is unchanged as shown in Figure 4b. The *ΔR* values of different LM‐HCFs are small, which can be ignored, leading to the resistance change rate depending mainly on the original resistance. According to the equation $R_{0}=\rho \frac{l_{0}}{A_{2}}$ (where *R_0_* is the resistance, ρ is the electrical resistivity of liquid metal, which is regarded as constant, *l_0_* is the helical length, *A_2_* is the cross‐sectional area of the helical), then $\Delta R/R_{0}=\frac{\Delta R\cdot A_{2}}{\rho \cdot l_{0}}$. With Q_out_/Q_in_ increases, the *l_0_* increases and *A_2_* decreases, resulting in Δ*R*/*R*_0_ decreases. A detailed discussion of the conductivity is in the Supporting Information. The results demonstrated that the LM‐HCFs with a smaller diameter of internal helical structure exhibit better insensitivity to conductivity under strain. The LM‐HCF exhibited a minuscule variation of Δ*R*/*R*_0_, only a 1.6% change of 100% strain. Even stretching to 600%, the best performance of strain‐insensitive conductivity is the Δ*R*/*R*_0_ value of 30%. After the calculation, a high Q value can be obtained, as shown in Figure 4c. Due to the Poisson effect of the elastic outer layer and the incompressibility of the liquid metal, at the beginning of the strain, the liquid pressure resists the shrinkage of the fibers, which may lead to a temporary increase in the inner diameter. Under continuous stretching, the axial tension preferentially overcomes the bending energy of the spiral, causing the helical structure inside the fibers to gradually unravel, corresponding to stage 1. As a result, the liquid pressure rises slowly, and the inner diameter change is small. After the helix is fully deployed, the liquid pressure cannot resist the fiber contraction, resulting in a smaller inner diameter, corresponding to stage 2. Therefore, it can be concluded that the inner diameter initially increases and subsequently decreases, leading to negative values of ΔR and Q at the initial strain, which is consistent with the results depicted in Figure 4c and Figure S11 (Supporting Information). The Q value of LC‐HCF is 62.5 at 100% strain. Compared with references as shown in Figure 4d,e and Table S1, the LM‐HCFs show unparalleled advantages in electrical conductivity, stretchability, and conductance stability.^[^ ^14^, ^22^, ^40^, ^41^, ^42^, ^43^, ^44^, ^45^, ^46^, ^47^ ^]^ When it is manually stretched, it was found that its current stability is excellent, the current change is only 80% with stretching 1300% as shown in Figure S12 (Supporting Information). To further investigate its strain‐insensitive conductivity, 10 000 cycles of 50% and 100% stretching are performed as shown in Figure 4f and Figure S13 (Supporting Information). The resistance changes are tiny, with ΔR/R_0_ of less than 5% and 10%, respectively. Even 20 cycles of 50%, 100%, and 200% stretching, ΔR/R_0_ is less than 20% (Figure S14, Supporting Information). Furthermore, benefiting from the high‐elasticity characteristics of the fiber cortex and the internal helical conductive pathway, when this fiber undergoes deformations such as bending, twisting, or compression, it primarily responds to external stresses through mechanisms including the bending, uncoiling, tightening, and unfolding of the helical structure. This sophisticated structural design effectively prevents significant alterations to the conductive pathway, thereby minimizing changes in electrical resistance, as shown in Figure 4g–i.

**Figure 5:**
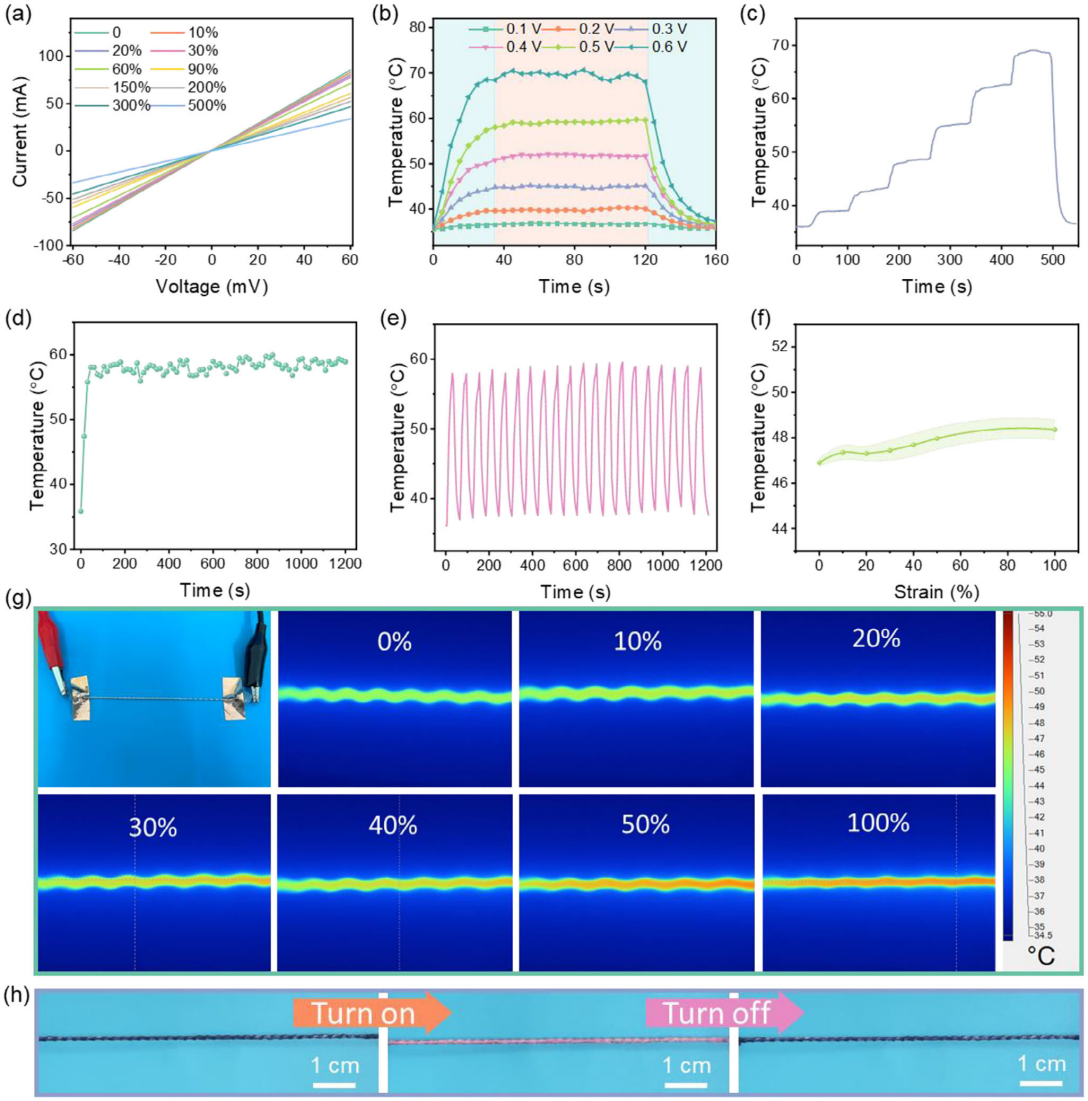
a) The *I–V* curves of LM‐HCF under different strains, b) Joule heating performance under different voltages, c) Joule heating performance under increasing voltage, d) Long‐term temperature curve of LM‐HCF at a voltage of 0.5 V for 1200 s, e) Cycle heating‐cooling performance of LM‐HCF at a voltage of 0.5 V, f) Heating performance of LM‐HCF at a voltage of 0.3 V under stretching, g) The corresponding image and infrared thermal images of (f), (h) Images of the electro‐thermochromic fiber at a voltage of 0.5 V.

According to Joule's law (*Q* = *U^2^t/R*, where *Q* is Joule heat, *U* is applied voltage, t is the operating time, and R is resistance), the high conductivity of LM‐HCF endows it with excellent Joule heating performance, which has potential application in personal thermal management devices such as thermal therapy and self‐heating clothing. The current‐voltage (*I–V*) curves of LM‐HCF in different strains are shown in **Figure** **5**a, which obey Ohm's law, demonstrating the stable electrical properties. Figure 5b exhibits the surface temperature of LM‐HCF at low applied voltages from 0.1 to 0.6 V. It can also be found that a higher applied voltage results in a higher surface temperature. The surface temperature gradually increases from room temperature to 70 °C at a low voltage of 0.6 V and quickly reaches equilibrium. Figure 5c shows that the temperature gradient rises by adjusting the voltage, indicating the controllability of the Joule heating property of LM‐HCF. To evaluate its stability, a voltage of 0.5 V was applied for a long time of 1200 s and heating‐cooling cycles, as depicted in Figure 5d,e, respectively. The LM‐HCF maintained a stable heating temperature of ≈58 °C in 1200 s and 20 cycles of heating‐cooling, indicating excellent heating stability and reliability for practical thermal management applications. Furthermore, the Joule heating performance of LM‐HCF was evaluated under strain with an applied voltage of 0.3 V, as shown in Figure 5f,g. It can be observed that the temperatures maintain ≈47–48 °C and increase slightly upon stretching, which is presumably ascribed to the stable resistance of LM‐HCF. The mechanism for generating the surface temperature of the LM‐HCF fiber involves two key processes: First, the liquid metal in the core layer produces Joule heat under the applied voltage, and then the heat is transferred from the liquid metal to the fiber sheath. According to Joule's law, an increase in resistance will lead to a decrease in Joule heat. When the LM‐HCF fiber is stretched, the variation in its electrical resistance is negligible, and correspondingly, the change in the surface temperature of the liquid metal can also be considered insignificant. However, the stretching process causes the fiber to become thinner in diameter, reducing the distance for heat conduction and thus decreasing the thermal resistance, which ultimately leads to a slight temperature increase. Additionally, thermochromic dyes can be coated onto fiber to endow it with electro‐thermochromic properties. As shown in Figure 5h and Video S6 (Supporting Information), when a voltage of 0.5 V is applied, the fiber rapidly turns pink due to the thermally induced color change of the dye. After the voltage is turned off, the color reverts to its original black state. The fiber was placed on a heating stage where the temperature was gradually increased from 50 to 170 °C, with each temperature held for 30 s. As exhibited in Figure S15 (Supporting Information), the fiber retains its complete structure without any liquid metal leakage throughout the process. Until the temperature reaches 170 °C, the fiber starts to melt and fracture, indicating its upper thermal stability limit. In addition, a prolonged heating test was conducted at a constant temperature of 60 °C to assess stability under sustained thermal exposure, as shown in Figure S1 6 (Supporting Information). It can be found that the fiber maintained its intact shape after 16 h of heating at 60 °C. It also keeps a good stretchability without liquid metal leakage, as shown in Figure S16i (Supporting Information), further confirming its robust stability under prolonged temperature conditions. These results demonstrate the significant potential of LM‐HCF in multifunctional wearable electronics.

**Figure 6:**
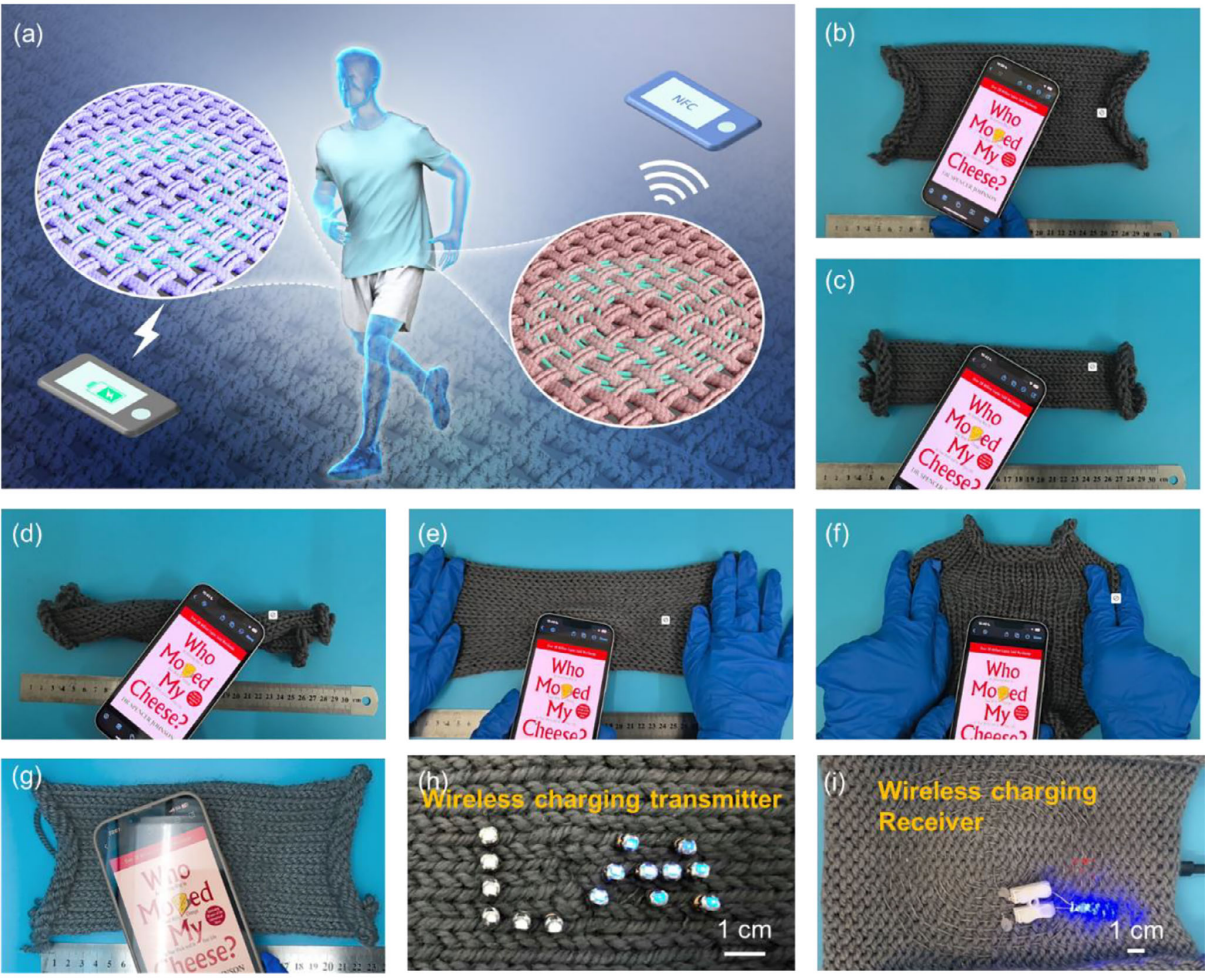
a) Schematic illustration of the flexible NFC device and wireless charging device, where the fiber is woven into coils. Photos of the NFC for label identification under different deformations: b) normal working, c) folding, d) twisting, e) longitudinal stretching, f) transverse stretching. g) 4000 bends. The image of fabric with LM‐HCF coil as the wireless charging h) transmitter and i) receiver to light up the LED.

The LM‐HCF was sewn into fabric in a circular pattern to fabricate a wireless NFC antenna as shown in **Figure** **6**a and S1 7(Supporting Information), which is beneficial to convenient and comfortable wireless communication. The fiber has a length of 230 cm and an electrical resistance of 20 Ω, significantly outperforming conductive textiles in terms of electrical conductivity. To evaluate the recognition performance of this NFC antenna, a series of deformations were applied as illustrated in Figure 6b–f. It can be found that regardless of whether the antenna was stretched transversely, stretched longitudinally, folded, or twisted, the fabric‐based NFC antenna could be readily and rapidly recognized. The corresponding recognition processes were recorded and shown in Video S7 (Supporting Information). The S11 magnitude of the fabric NFC antenna under different deformations and stretching was measured as shown in Figure S18 (Supporting Information). The resonance frequency and operational bandwidth exhibit a slight change as stretching increases, indicating good stability of this fabric NFC antenna. To further evaluate the durability of this fabric NFC antenna, 90° cyclic bending tests were conducted. As shown in Figure 6g, the NFC antenna remained functional and recognizable even after 4000 bending cycles, demonstrating excellent durability and promising prospects for wearable electronics. The details are presented in Figure S19 and Video S8 (Supporting Information). What's more, the LM‐HCF can be employed in a wireless charging device. As displayed in Figure 6h,i and Figure S20 (Supporting Information), it was woven into a fabric to be a transmitter and receiver for LED lighting. The detailed charging process is shown in Video S9 (Supporting Information). These experiments validate the feasibility of LM‐HCF as a flexible, textile‐compatible medium for signal transmission, highlighting its promising role in next‐generation flexible wearable electronics.

## 3 Conclusion

In summary, this work developed a highly elastic hollow fiber with a helical channel inside via coaxial wet‐spinning technology, which achieves an impressive elongation at break of ≈1440%. The injection of liquid metal into this channel endowed the fiber with an exceptional conductivity of 1.94 × 10^5^ S m^−1^. The helical structure ensures stable conductivity, with a resistance change of less than 1.6% under 100% strain and only 30% even when stretched to 600%. Additionally, the fiber demonstrates remarkable stability under bending, twisting, and pressing conditions. The PU sheath provides waterproof properties and allows the fiber to function effectively in aquatic environments. Furthermore, these fibers can be woven into fabrics, exhibiting superior performance in applications such as joule heaters, near‐field communication, and wireless charging, indicating great promise for practical use in wearable electronics, smart electronics, and human‐machine interfaces. This research provides a new strategy and material basis for developing flexible electronics with enhanced comprehensive performance.

## 4 Experimental Section

Polyurethane (PU 80A) was supplied by Shanghai Junen Plasticizing Co., Ltd. (China). N, N‐dimethylformamide (DMF) was purchased from Sinopharm Chemical Reagent Co., Ltd. Dual‐channel micro‐injection pump was provided by Shandong Zibo Guanjie Electronic Technology Co., LTD. Liquid metal (Ga/In/Sn/Zn) was purchased from Dongguan Dingguan Metal Technology Co., LTD. The coaxial needles of 30/17 G and 30/14 G were purchased from the Taobao website. The wireless charging transmitting module (with an input voltage of 5 V and a maximum charging power of 3 W), wireless bulbs (4.3 mm × 4.3 mm, with a voltage of 2.8–3.4 V and a current of 5 mA), and an LED (with a rated voltage of 3.0–3.2 V and a rated current of 20 mA) were purchased from Taobao website.

### Preparation of Helical Channel Fiber by Wet Spinning

PU was dissolved in DMF, and the mixture was stirred for 8 h at room temperature and subjected to ultrasonic defoaming for 4 h to obtain a uniform and transparent PU spinning solution. The outer phase of 14–20 wt.% PU solution and the inner phase of pure water were extruded from the coaxial needle (Figure S1a, Supporting Information) into a water coagulation bath. Coaxial needles of other sizes could be assembled using a flat needle, a Y‐joint of the indwelling needle, and a capillary tube, as shown in Figure S1b (Supporting Information). Different internal structures of the fiber could be achieved by adjusting the flow rate ratio of the inner and outer phases (Q_in_/Q_out_) using a dual‐channel micro‐injection pump.

### Preparation of Highly Elastic and Conductance‐Stable Fiber

The raw fiber contained a lot of water in the fiber channel. After removal, the liquid metal was injected into the helical channel of the fiber to endow it with high conductivity. The fiber ends were subsequently sealed with hot‐melt adhesive to form an encapsulated elastic conductive fiber, followed by drying at room temperature.

### Preparation of the NFC Device and the Wireless Charging Device

A 230 cm LM‐HCF was manually sewn into a 16 × 16 cm knit fabric. The LM‐HCF was arranged in a coil configuration with an outer long diameter of 12 cm and short diameter of 10 cm, a coil spacing of 0.5 cm, and 12 coil turns. The two ends of the LM‐HCF were connected to the chip, which was taken from a commercial NFC. The schematic diagram of the NFC antenna structure is presented in Figure S21 (Supporting Information). When the chip was replaced with a commercial wireless transmission module, the system could function as a wireless transmitter. When substituting the chip with an LED, the system acted as a receiver to light up the LED. The reader was set up by adding an NFC tag via Shortcuts on iPhone 13, with the command to open a Freeform board displaying the picture of “Who Moved My Cheese”. The resonant frequency was measured by a vector network analyzer (ZNB 20, Rohde & Schwarz).

### Calculation of the Conductivity of LM‐HCF

The electrical conductivity of the LM‐HCF is calculated by the equation:

(1) $$\sigma =\frac{L_{0}}{R_{0}\cdot A_{1}}$$

where *R_0_* is resistance, *A_1_* is the whole area of LM‐HCF, and *L_0_* is the length of LM‐HCF.

The quality factor (Q) can be calculated as follows:

(2) $$Q=\frac{\left(L_{x}-L_{0}\right)/L_{0}}{\left(R_{x}-R_{0}\right)/R_{0}}$$

where L_0_ is the length of the fiber, L_x_ is the length of the fiber under stain. R_0_ is the resistance of the fiber, and R_x_ is the resistance of the fiber under stain. The fiber strain (ε_*L*_) can be calculated as:

(3) $$\varepsilon_{L}=\frac{L_{X}-L_{0}}{L_{0}}$$

The helical path stain can be calculated as:

(4) $$\varepsilon_{l}=\frac{l_{x}-l_{0}}{l_{0}}$$

the *l*_0_ is the helical length, *l_x_* is the helical length under strain. The resistance of fiber under strain can be described as:

(5) $$R_{x}=\rho \frac{l_{x}}{\pi {\left(d_{x}/2\right)}^{2}}$$

while the ρ is the electrical resistivity of liquid metal, which is regarded as constant, *l_x_* is the helical length under strain, and *d*_x_ is the diameter of the helical cross‐section under strain. The liquid metal is an incompressible liquid, so the volume is constant:

(6) $${d_{0}}^{2}l_{0}={d_{x}}^{2}l_{x}$$

where *l_0_* is the original helical length, and *d_0_* is the original diameter of the helical cross‐section. After the calculation, Q can be expressed as:

(7) $$Q=\frac{\varepsilon_{L}}{{\varepsilon_{l}}^{2}+2\varepsilon_{l}}$$

### Measurement

The fiber morphology was observed using a field emission scanning electron microscope (FESEM, 7800F, JEOL) and a 3D ultra‐depth of field microscope. The mechanical property was measured by an Instron universal testing machine (Instron 5970, Instron Corporation). The electrical conductivity was tested by a digital source meter (Keithley, 2450). The strain and cyclic stretching tests were conducted using self‐built equipment consisting of a tension tester (TM‐550, Huicen Technology) and a digital source. A multifunctional flexible bending tester (FB‐240, Huicen Technology) was used to measure the resistance change of the fiber under bending and twisting. The reader was set up by adding an NFC tag via the Shortcuts app on iPhone 13, with the command to open a Freeform board displaying the picture of “Who Moved My Cheese”. The resonant frequency was measured by a vector network analyzer (ZNB 20, Rohde & Schwarz).

## Conflict of Interest

The authors declare no conflict of interest.

## Supporting information



Supporting Information

Supplemental Video 1

Supplemental Video 2

Supplemental Video 3

Supplemental Video 4

Supplemental Video 5

Supplemental Video 6

Supplemental Video 7

Supplemental Video 8

Supplemental Video 9

## Data Availability

The data that support the findings of this study are available from the corresponding author upon reasonable request.
