# Supplementary material for: A Liquid Metal‐Embedded Sheath‐Core Fiber with Internal Helical Structure for Strain‐Insensitive Electronics
Source: Adv Sci (Weinh). 2025 Jul 21;12(39):e09547. doi: 10.1002/advs.202509547 (PMC12533390; doi:10.1002/advs.202509547)
Supplement: Supplementary file 1 — Supporting Information [file ADVS-12-e09547-s010.docx]

***Supplementary Materials***

**A Liquid Metal-Embedded Sheath-Core Fiber with internal helical structure for strain-insensitive Electronics**

Mengying Luo^1#^, Wanru Wei^1#^, Qiye Guo^1^, Weibing Zhong^1^, Kangyu Jia^1^, Kangqi Chang^2^, Ying Lu^1^, Mufang Li^1, *^, Dong Wang^1, *^

1. Key Laboratory of Textile Fiber and Products, Ministry of Education, Wuhan Textile University, Wuhan 430200, China
2. Institute of Technology for Future Industry, Shenzhen Institute of Information Technology, Shenzhen 518172, China

*Corresponding authors: E-mail: limufang223@126.com

E-mail: [wangdon08@126.com](mailto:wangdon08@126.com)

#These authors contributed equally to this work.

For the fabrication of a transparency needle, a 34 G needle was first inserted into the straight channel end of the Y-connector. The joint was reinforced with hot-melt adhesive to guarantee a reliable and robust connection. Subsequently, a capillary glass tube with a diameter of 1 mm was inserted through the front end of the Y-connector, as depicted in Fig. S1(b).
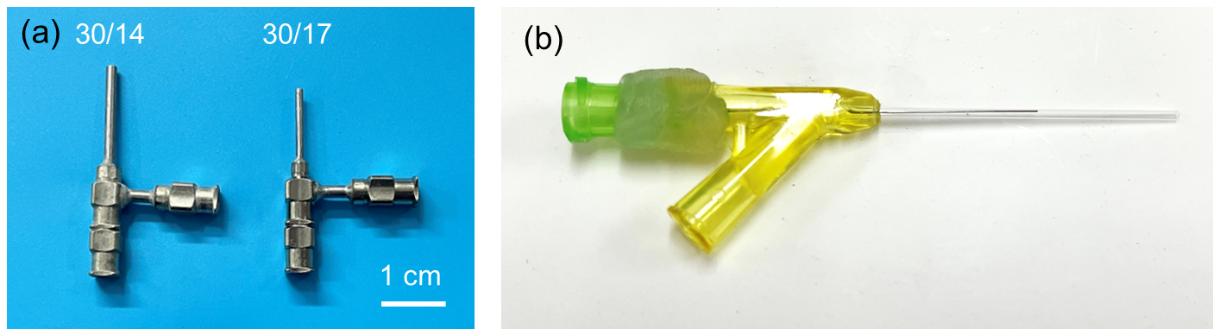


Fig. S1(a) The image of the coaxial needle of 30/14 G and 30/17 G, (b) Image of the assembled coaxial needle


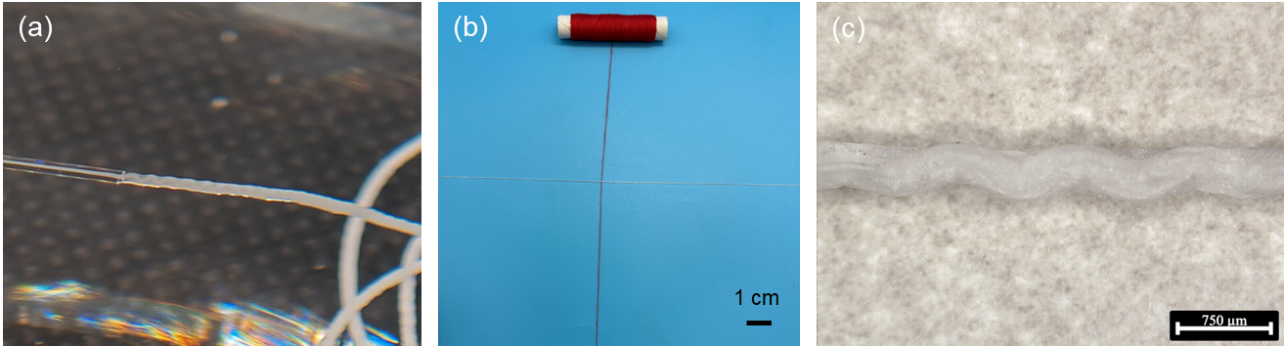


Fig. S2 (a) Photo image of the wet spinning of the HCF using a 30/21 G needle gauge, (b) Photo image of the HCF and sewing thread, (c) Microscope image of the HCF


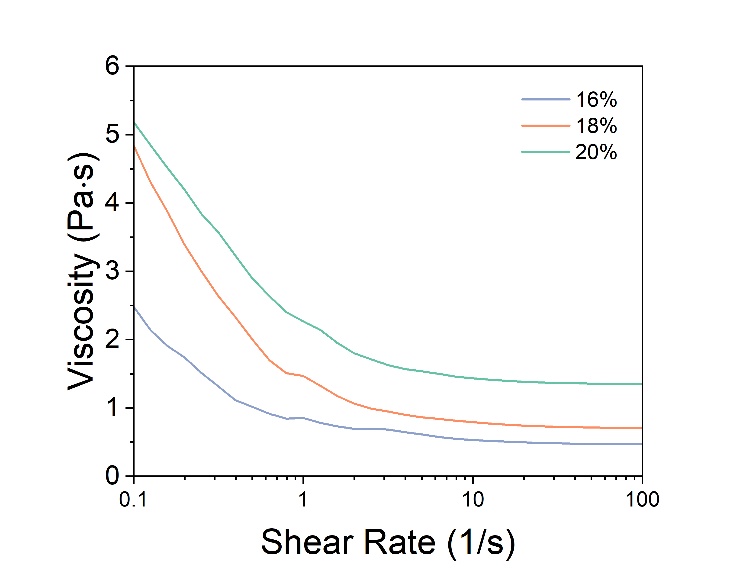


Fig.S3. Viscosity-shear rate curves of different PU concentrations.


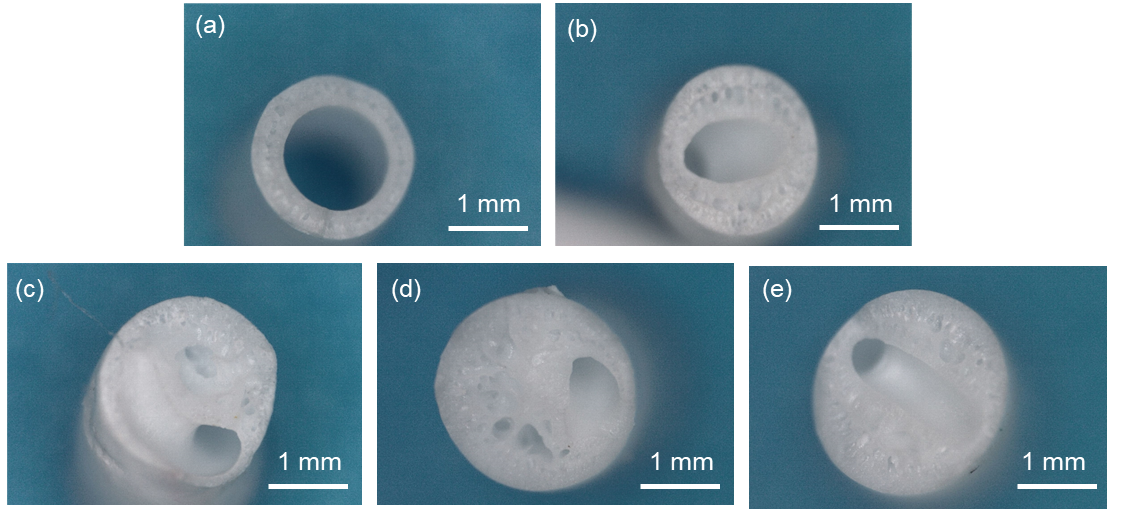


Fig. S4. Cross-sectional images of the fiber fabricated with different Q_out_/Q_in_, needle size 30/14, (a) 16:4, (b) 20:4, (c) 24:4, (d) 28:4, (e) 32:4


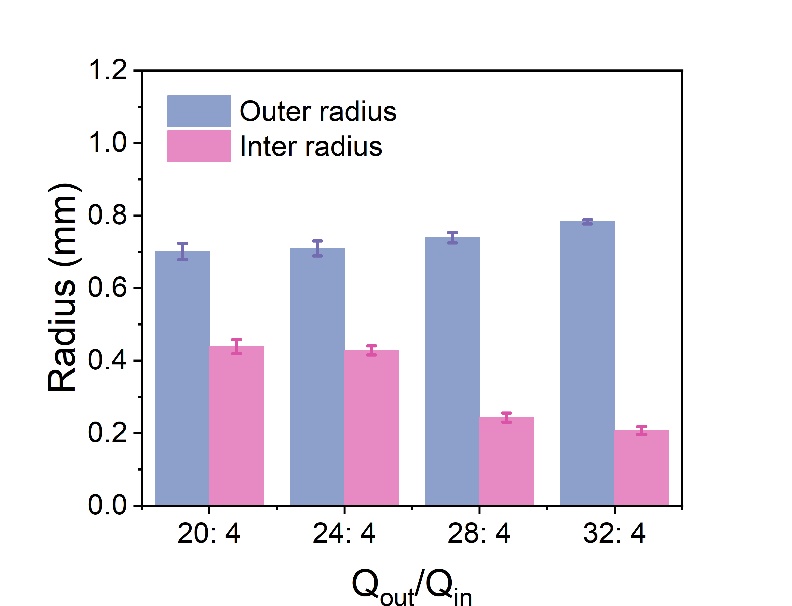


Fig. S5 Relationship between the radius of the fiber and Qout/Qin, needle size 30/14, CPU=16 wt%


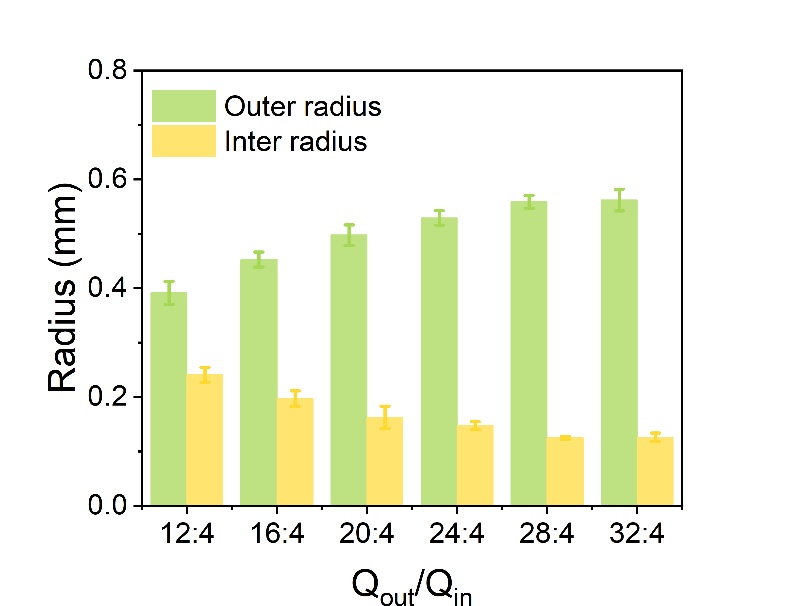


Fig. S6 Relationship between the radius of the fiber and Qout/Qin, needle size 30/17, CPU=20 wt%


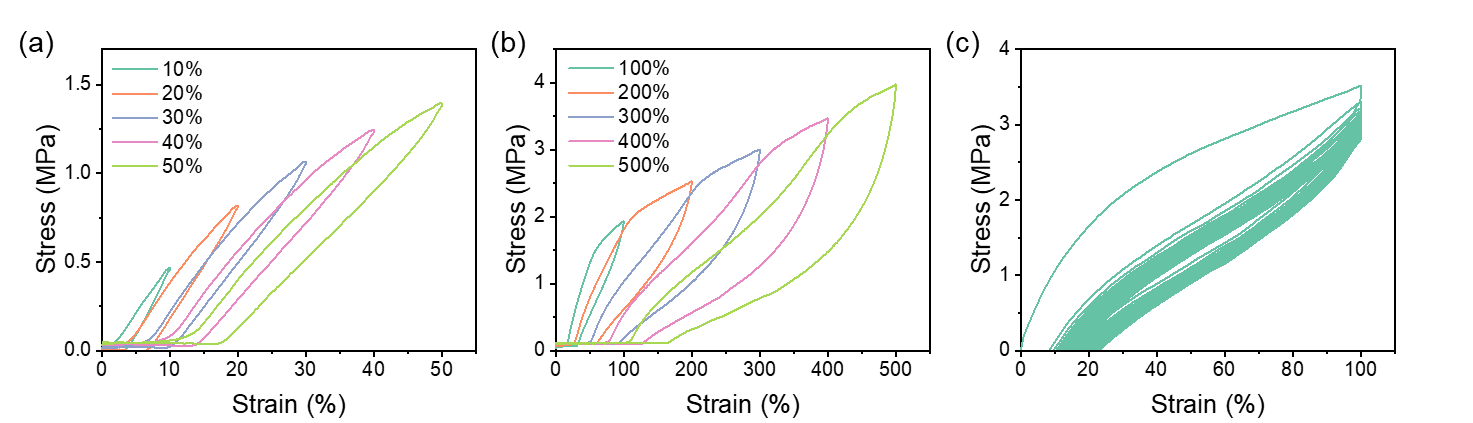


Fig. S7. (a) (b) Stress-strain curves of HCF under different strains, (c) Stress-strain curves of HCF under 100% for 100 cycles


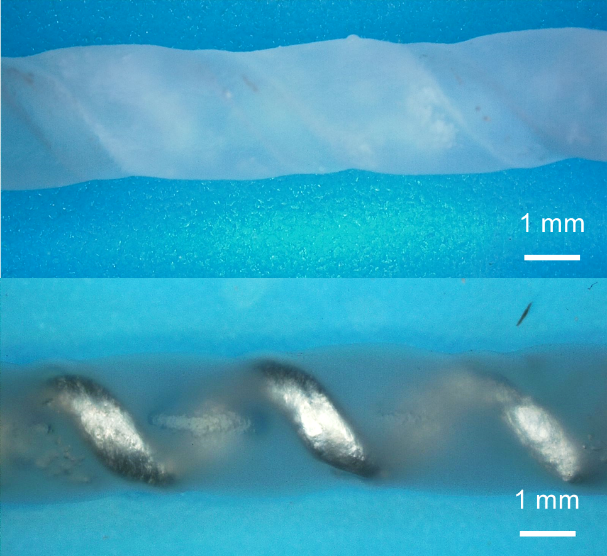


Fig. S8 Image of the HCF and LM-HCF after immersing in dichloromethane

To study the barrier property of polyurethane (PU) against liquid metals, a 25-cm-long liquid metal-filled hollow fiber (LM-HCF) was subjected to pressing, twisting, and stretching tests, and its morphologies were continuously recorded for 10 days. As shown in Fig. S10, the fiber surfaces remained clean without obvious liquid metal leakage. The Supporting video 4 captures the detailed process, demonstrating that wiping the fiber surface left the tissue completely clean, further confirming no liquid metal seepage. These results highlight the excellent barrier property of the PU layer.
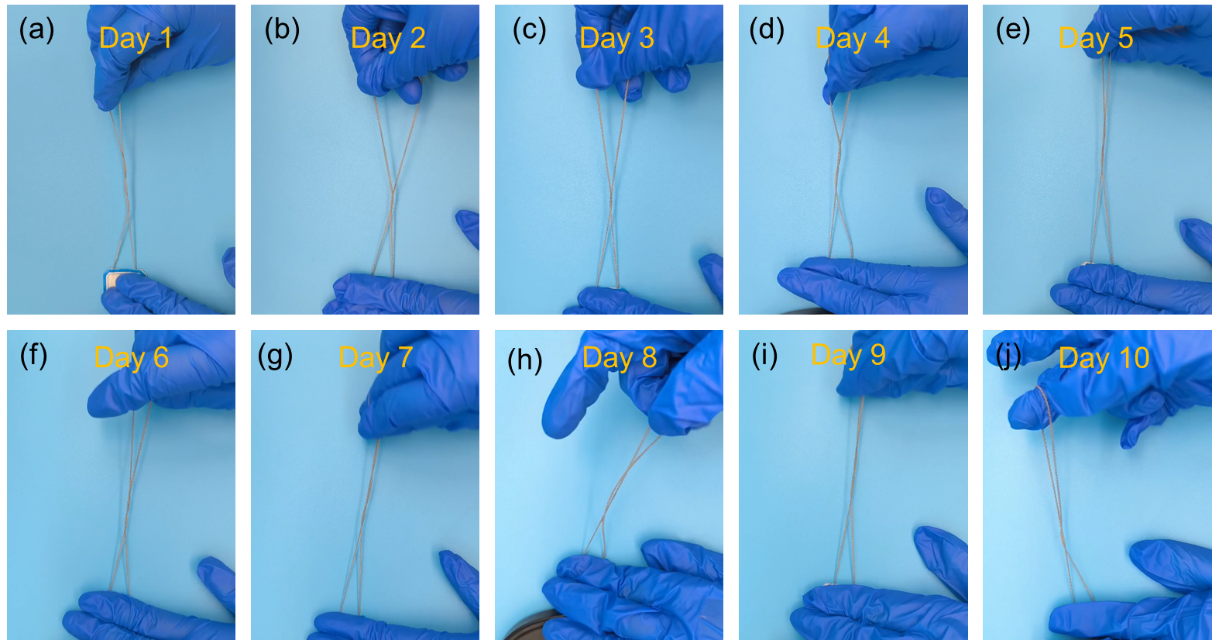


Fig. S 9. The morphological image of LM-HCF under deformation from day 1 to day 10.


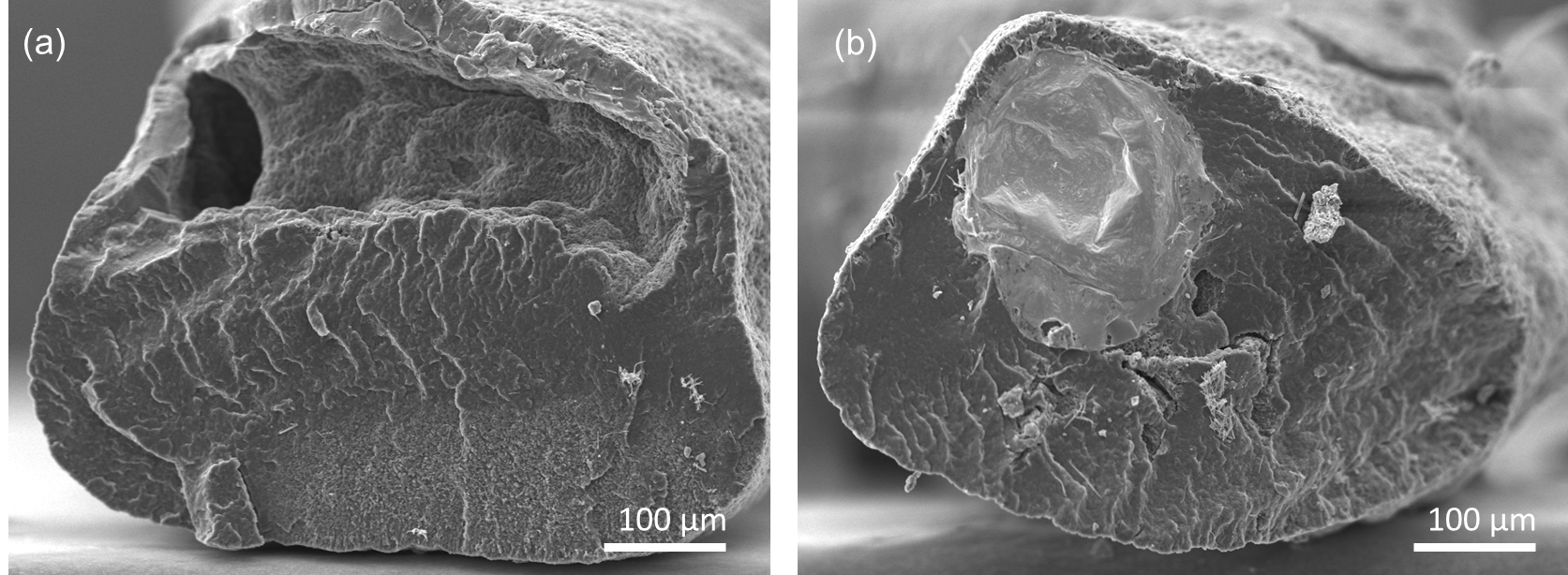


Fig. S10 Cross-sectional SEM image of HCF and LM-HCF

The effect of the Q_out_/Q_in_ on the electrical conductivity of the LM-HCF

Theoretically, with the increase in external flow rate (Q_out_), the time required to form fibers of a specific length decreases. This leads to a reduction in internal flux, thereby causing the hollow space to decrease. The conductivity of the fiber originates from the inner helical liquid metal, then the resistance value can be calculated by the equation $R_{in}=\rho\frac{l_{helix}}{A_{in}}$. For the entire fiber, its electrical conductivity can be calculated as follows: $\sigma=\frac{L_{out}\cdot A_{in}}{l_{helix}\cdot A_{out}\cdot\rho}$. Disregard of the swelling or shrinkage of the fiber in formation process, the inner helical length and the fiber length can be calculated by the equations $l_{helix}=\frac{Q_{in}}{A_{in}}$ and $L_{out}=\frac{Q_{out}+Qin}{A_{out}}$, respectively. Then it can be calculated as $\sigma=\frac{1}{\rho}\cdot\left( \frac{A_{in}}{A_{out}} \right)^{2}\cdot\left( \frac{{Q_{out}+Q}_{in}}{Q_{in}} \right)$. According to a equation^8^ $\frac{D_{in}}{D_{out}}=\left( 1-\left( \frac{Q_{out}}{Q_{out}+Q_{in}} \right)^{0.5} \right)^{0.5}$, it can be obtained $\left( \frac{A_{in}}{A_{out}} \right)^{2}=\left( 1-\left( \frac{Q_{out}}{Q_{out}+Q_{in}} \right)^{0.5} \right)^{2}$, and the σ can be described as $\sigma=\frac{1}{\rho}\left( 1-\left( \frac{Q_{out}}{Q_{out}+Q_{in}} \right)^{0.5} \right)^{2}$. It can be found that when Q_out_ increases, the σ decreases.


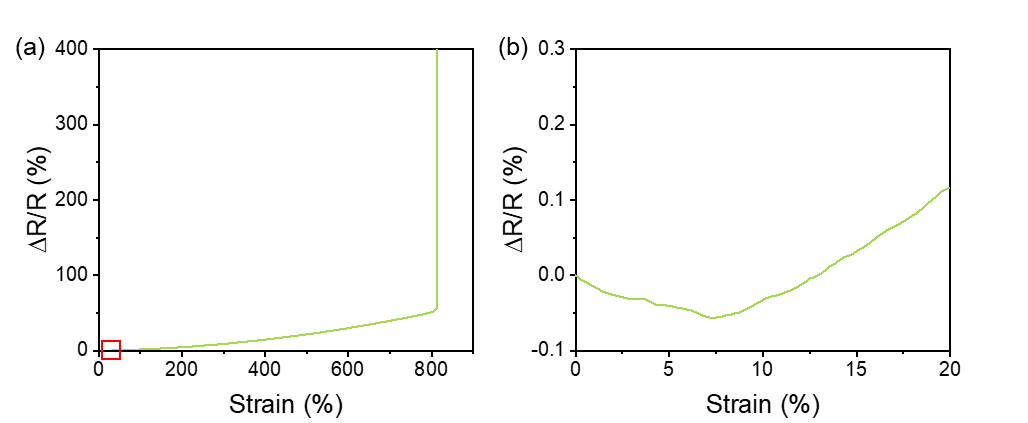


Fig. S11 Stain-dependent resistance changing rate of LM-HCF (32:4) and magnification.


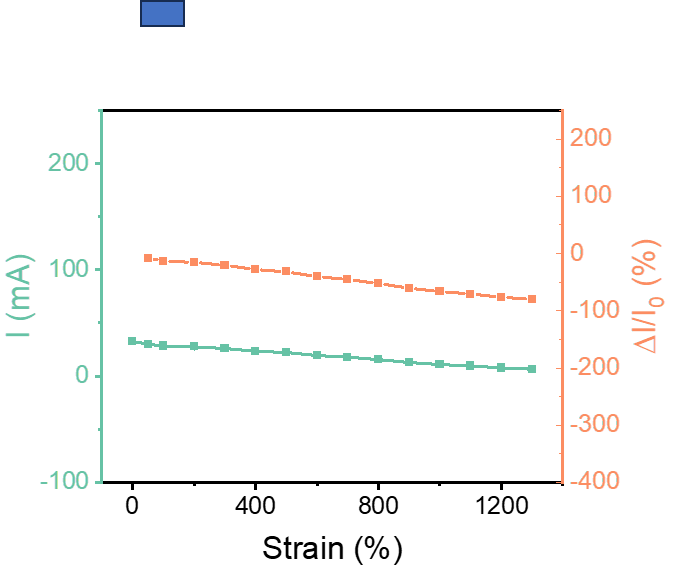


Fig. S12 Current and current change rates of the LM-HCF under strain.

Fig. S13. Resistance change rates of the LM-HCF under 10000 cycles of 100% stretching.


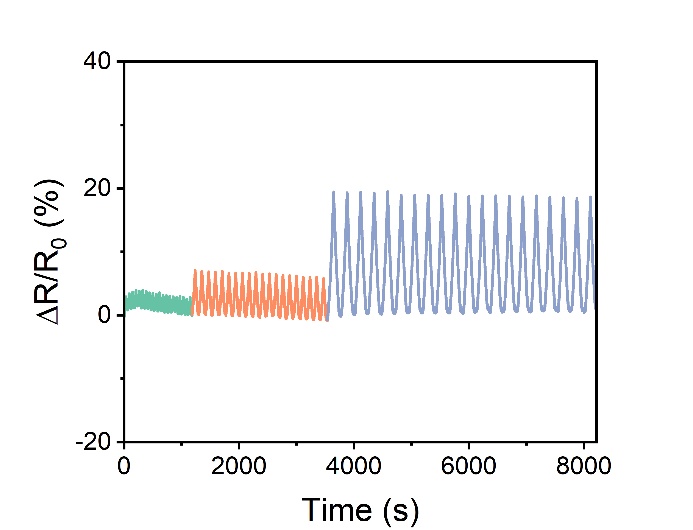


Fig. S14. Resistance change rates of the LM-HCF under 20 cycles of 50%, 100%, 200% stretching.

Table S1 Comparison of the electrical performance between LM-HCF and the reported conductance-stable materials.

| **Shape** | **Strategy** | **Conductive Material** | **σ (S/m)** | **Encapsulation** | **Max Strain** | **Strain-Insensitivity Performance** | | | **Ref.** |
| --- | --- | --- | --- | --- | --- | --- | --- | --- | --- |
|  |  |  |  |  |  | Strain | ΔR/R_0_ | Q |  |
| Fiber | ***Helic*** | ***Liquid Metal*** | ***1.94×10^5^*** | ***Yes*** | ***1300%*** | ***100%*** | ***1.6%*** | ***62.5*** | ***This work*** |
|  |  |  |  |  |  | ***600%*** | ***30%*** | ***20*** |  |
|  |  |  |  |  |  | ***800%*** | ***51%*** | ***15.69*** |  |
|  | Tortuous Serpentine | Liquid Metal | 4.35×10^4^ | Yes | 1170% | 200% | 4% | 50 | ^1^ |
|  | Buckling | MWCNTs/AgNWs | 2×10^4^ | No | 250% | 100% | 10% | 10 | ^2^ |
|  | Wrinkling | Au | 461 | No | 900% | 100% | 50% | 2 | ^3^ |
|  | Helic | Silver | 873 | Yes | 300% | 275% | 6% | 47.4 | ^4^ |
|  | Helic | PVA/PEDOT: PSS | 14700 | Yes | 500 | 100% | 5% | 20 | ^5^ |
|  | Worm Shape | Graphene | 124 | No | 1010% | 220% | 10% | 11.26 | ^6^ |
|  | Solid-Liquid Bicontinuous | LITFSI | 0.04 | No | 750% | 200% | 7% | 28.57 | ^7^ |
|  | Buckling | PPy | 238 | No | 900% | 900% | 350% | 2.6 | ^8^ |
|  |  |  |  |  |  | 200% | 18% | 10.9 |  |
|  | Winkling | PPy | 100 | No | 600% | 600% | 66% | 9.09 | ^9^ |
|  | Helic | Carbon Tube | ~300 | No | 285% | 25% | 3.2% | 7.81 | ^10^ |
|  | Helical Microchannel | Ionic Liquids | - | Yes | 230% | - | - | 1 | ^11^ |
|  | Helical | Carbon Fiber | - | Yes | 300% | 100% | 9% | 11.1 | ^12^ |
|  |  |  |  |  |  | 200% | 30% | 6.67 |  |
|  | Buckling | Carbon Nanotube | - | No | 1320% | 1000% | 5% | 200 | ^13^ |
|  | Buckling | PEDOT: PSS | 9500 | Yes | 700% | 680% | 4% | 170 | ^14^ |
| Film | Mesh | Liquid Metal | 1.8×10^6^ | No | 2000% | 1800% | 4.1% | 441 | ^15^ |
|  | Regular Mesh | Ti_3_C_2_T_x_ Mxene/Ag Nws | 33.3 Ω/m^2^ | Yes | 90% | 60% | - | - | ^16^ |
|  | Wrinkled Structure | Mxene | - | Yes | 100% | - | - | 4 | ^17^ |
|  | Winkled | Au | - | No | 100% | 80% | 40% | 1.34 | ^18^ |
| Strip | Helical | Copper Wire |  | Yes | 100% |  |  | - | ^19^ |
| Sponge | Sponge | Gold Nanowire | 1500 | No | 340% | 50% | 17.3% | 2.89 | ^20^ |
|  |  |  |  |  |  | 100% | 83.3% | 1.2 |  |

**
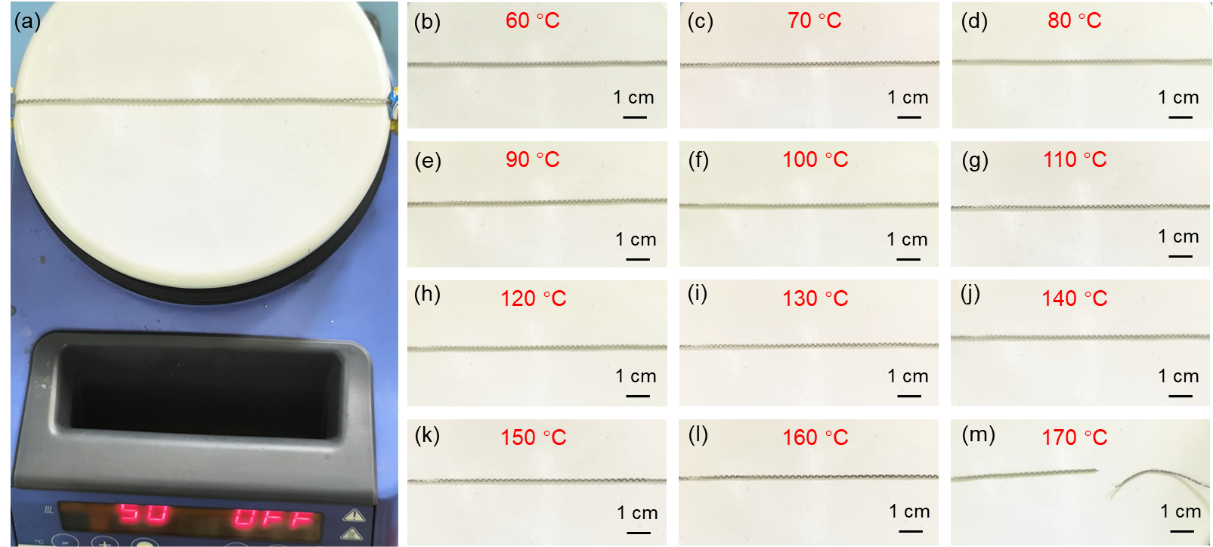
**

Fig. S15 Image of the LM-HCF on a heating stage with an increasing temperature

**
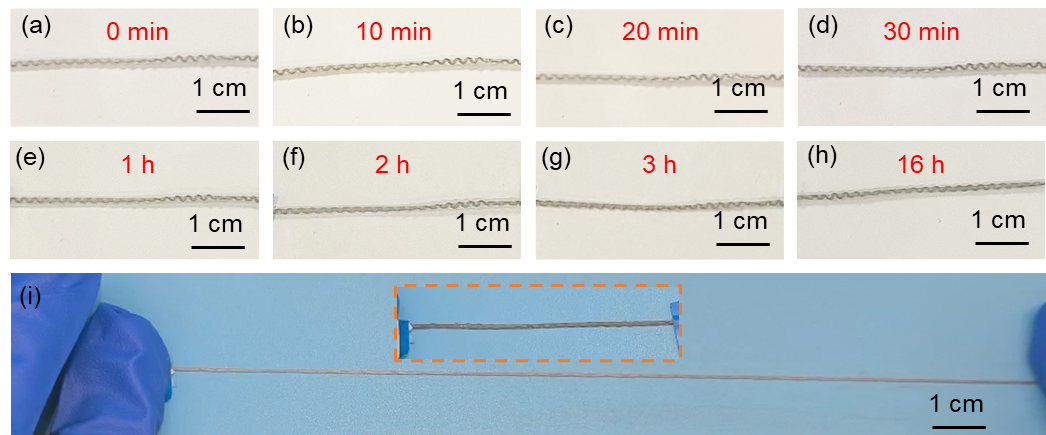
**

Fig. S16 (a)-(h) Image of the LM-HCF on a heating stage with increasing time, (i) Image of the stretching of the LM-HCF after heating for 16 h


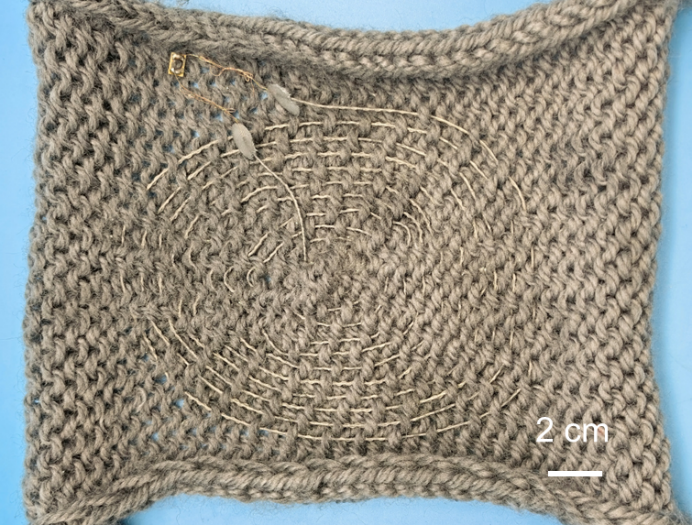


Fig. S17 Photo image of the NFC device


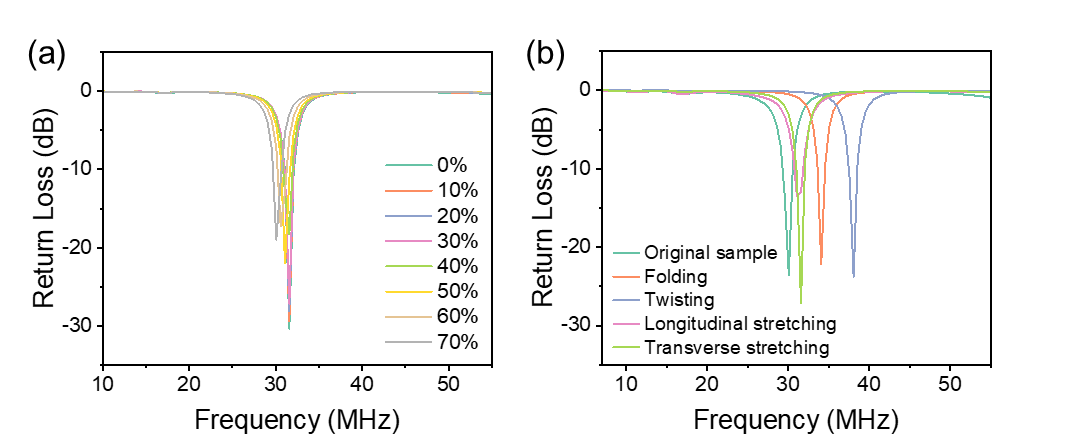


Fig. S18. Return loss of the fabric NFC under (a) strain, (b) deformations.

**
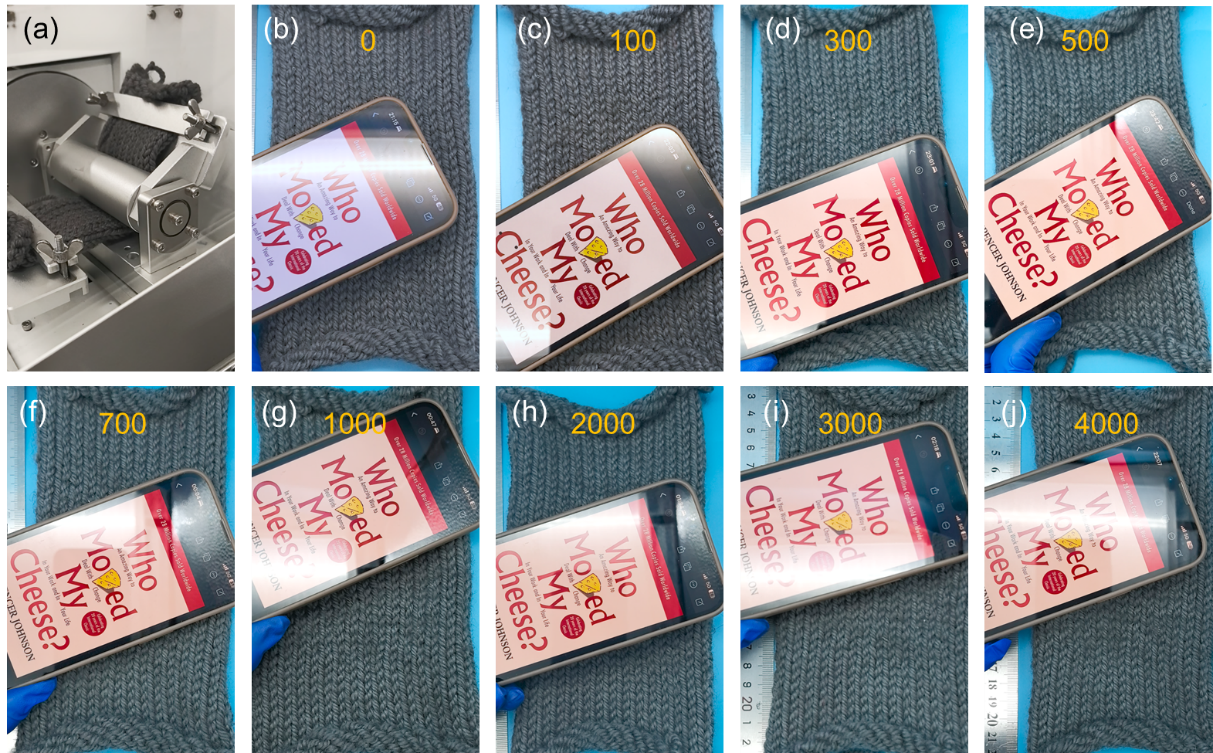
**

Fig. S19 Photo image of (a) bending of the NFC fabric, NFC for label identification after (b) 0, (c) 100, (d) 300, (e) 500, (f) 700, (g) 1000, (h) 2000, (i) 3000, (j) 4000 bending cycles


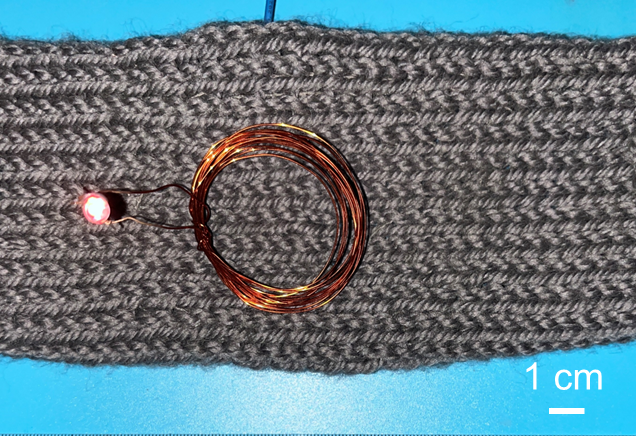


Fig. S 20 The image of fabric using the LM-HCF coil as a wireless charging transmitter to light up the LED


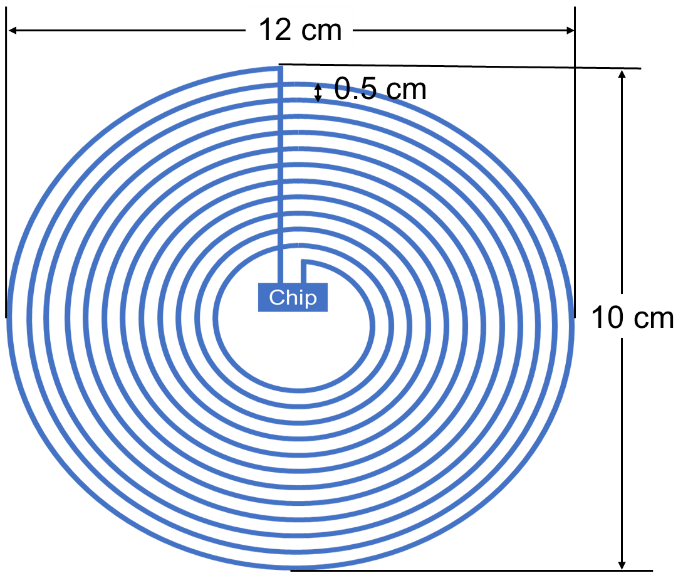


Fig. S21 Schematic diagram of NFC coil structure

**References**

1. Zheng, L.; Zhu, M.; Wu, B.; Li, Z.; Sun, S.; Wu, P., Conductance-stable liquid metal sheath-core microfibers for stretchy smart fabrics and self-powered sensing. *Sci Adv* **2021,** *7* (22).

2. Zhang, Y.; Zhang, W.; Ye, G.; Tan, Q.; Zhao, Y.; Qiu, J.; Qi, S.; Du, X.; Chen, T.; Liu, N., Core–Sheath Stretchable Conductive Fibers for Safe Underwater Wearable Electronics. *Advanced Materials Technologies* **2020,** *5* (1), 1900880.

3. Zhao, Y.; Dong, D.; Gong, S.; Brassart, L.; Wang, Y.; An, T.; Cheng, W., A Moss-Inspired Electroless Gold-Coating Strategy Toward Stretchable Fiber Conductors by Dry Spinning. *Advanced Electronic Materials* **2019,** *5* (1), 1800462.

4. Liu, Y.; Tang, Y.; Guo, X.; Qu, L.; Liu, Y.; Zhang, X.; Huang, T.; Xu, L.; Liu, H.; Tian, M., Template-Free and Stretchable Conductive Fiber with a Built-In Helical Structure for Strain-Insensitive Signal Transmission. *ACS Applied Materials & Interfaces* **2023,** *15* (39), 46379-46387.

5. Sun, T.; Liang, Y.; Ning, N.; Wu, H.; Tian, M., Strain-Insensitive Stretchable Conductive Fiber Based on Helical Core with Double-Network Hydrogel. *Advanced Fiber Materials* **2025**.

6. Sun, F.; Tian, M.; Sun, X.; Xu, T.; Liu, X.; Zhu, S.; Zhang, X.; Qu, L., Stretchable Conductive Fibers of Ultrahigh Tensile Strain and Stable Conductance Enabled by a Worm-Shaped Graphene Microlayer. *Nano Letters* **2019,** *19* (9), 6592-6599.

7. Ye, H.; Wu, B.; Sun, S.; Wu, P., A Solid–Liquid Bicontinuous Fiber with Strain-Insensitive Ionic Conduction. *Advanced Materials* **2024,** *36* (25), 2402501.

8. Li, Y.; Gao, Y.; Lan, L.; Zhang, Q.; Wei, L.; Shan, M.; Guo, L.; Wang, F.; Mao, J.; Zhang, Z.; Wang, L., Ultrastretchable and wearable conductive multifilament enabled by buckled polypyrrole structure in parallel. *npj Flexible Electronics* **2022,** *6* (1), 42.

9. Gao, Y.; Yu, L.; Li, Y.; Wei, L.; Yin, J.; Wang, F.; Wang, L.; Mao, J., Maple Leaf Inspired Conductive Fiber with Hierarchical Wrinkles for Highly Stretchable and Integratable Electronics. *ACS Applied Materials & Interfaces* **2022,** *14* (43), 49059-49071.

10. Shang, Y.; Li, Y.; He, X.; Zhang, L.; Li, Z.; Li, P.; Shi, E.; Wu, S.; Cao, A., Elastic carbon nanotube straight yarns embedded with helical loops. *Nanoscale* **2013,** *5* (6), 2403-2410.

11. Chen, S.; Liu, H.; Liu, S.; Wang, P.; Zeng, S.; Sun, L.; Liu, L., Transparent and Waterproof Ionic Liquid-Based Fibers for Highly Durable Multifunctional Sensors and Strain-Insensitive Stretchable Conductors. *ACS Applied Materials & Interfaces* **2018,** *10* (4), 4305-4314.

12. Nie, D.; Yan, Z.; Chen, J.; Zheng, Y.; Hu, X.; Ning, H.; Wu, Y.; Dai, J.; Chen, W.; Zhang, W.; Liu, X.-Y.; Lin, N., Strain-insensitive fiber sensors bioinspired by spider silk with a multilevel helical structure. *Chemical Engineering Journal* **2024,** *500*, 157489.

13. Liu, Z. F.; Fang, S.; Moura, F. A.; Ding, J. N.; Jiang, N.; Di, J.; Zhang, M.; Lepró, X.; Galvão, D. S.; Haines, C. S.; Yuan, N. Y.; Yin, S. G.; Lee, D. W.; Wang, R.; Wang, H. Y.; Lv, W.; Dong, C.; Zhang, R. C.; Chen, M. J.; Yin, Q.; Chong, Y. T.; Zhang, R.; Wang, X.; Lima, M. D.; Ovalle-Robles, R.; Qian, D.; Lu, H.; Baughman, R. H., Hierarchically buckled sheath-core fibers for superelastic electronics, sensors, and muscles. *Science* **2015,** *349* (6246), 400-404.

14. Zhou, J.; Tian, G.; Jin, G.; Xin, Y.; Tao, R.; Lubineau, G., Buckled Conductive Polymer Ribbons in Elastomer Channels as Stretchable Fiber Conductor. *Advanced Functional Materials* **2020,** *30* (5), 1907316.

15. Ma, Z.; Huang, Q.; Xu, Q.; Zhuang, Q.; Zhao, X.; Yang, Y.; Qiu, H.; Yang, Z.; Wang, C.; Chai, Y.; Zheng, Z., Permeable superelastic liquid-metal fibre mat enables biocompatible and monolithic stretchable electronics. *Nature Materials* **2021,** *20* (6), 859-868.

16. Gao, F.; Zhang, Z.; Zhao, X.; An, L.; Xu, L.; Xun, X.; Zhao, B.; Ouyang, T.; Kang, Z.; Liao, Q.; Zhang, Y., Highly conductive and stretching-insensitive films for wearable accurate pressure perception. *Chemical Engineering Journal* **2022,** *429*, 132488.

17. Yang, J.; Xu, Y.; Guo, Q.; Yin, F.; Yuan, W., Highly stretchable pressure sensors with wrinkled fibrous geometry for selective pressure sensing with minimal lateral strain-induced interference. *Composites Part B: Engineering* **2021,** *217*, 108899.

18. Chen, Z.; Zhou, R.; Huang, J.; Xu, H.; Li, Z.; Wang, Y.; Bao, R.; He, J.; Pan, C., Strain-Insensitive Pre-Stretch-Stabilized Polymer/Gold Hybrid Electrodes for Electrochemiluminescent Devices. *Advanced Functional Materials* **2024,** *34* (44), 2406434.

19. Zhao, Y.; Tan, Y. J.; Yang, W.; Ling, S.; Yang, Z.; Teo, J. T.; See, H. H.; Lee, D. K. H.; Lu, D.; Li, S.; Zeng, X.; Liu, Z.; Tee, B. C. K., Scaling Metal-Elastomer Composites toward Stretchable Multi-Helical Conductive Paths for Robust Responsive Wearable Health Devices. *Advanced Healthcare Materials* **2021,** *10* (17), 2100221.

20. Lin, F.; Wang, K.; An, T.; Zhu, B.; Ling, Y.; Gong, S.; Liu, S.; Cheng, W., Soft gold nanowire sponges for strain-insensitive conductors, wearable energy storage and catalytic converters. *Journal of Materials Chemistry C* **2021,** *9* (42), 15329-15336.
